# Simple Excel and ICD-10 based dataset calculator for the Charlson and Elixhauser comorbidity indices

**DOI:** 10.1186/s12874-021-01492-7

**Published:** 2022-01-07

**Authors:** Pärt Prommik, Kaspar Tootsi, Toomas Saluse, Eiki Strauss, Helgi Kolk, Aare Märtson

**Affiliations:** 1 Department of Traumatology and Orthopaedics, University of Tartu, L. Puusepa 8, 50406 Tartu, Estonia; 2 Traumatology and Orthopaedics Clinic, Tartu University Hospital, L. Puusepa 8, 50406 Tartu, Estonia; 3 Institute of Sport Sciences and Physiotherapy, University of Tartu, Ujula 4, 51008 Tartu, Estonia

**Keywords:** Charlson comorbidity index, Elixhauser comorbidity index, ICD-10, Comorbidity calculator, Research methodology

## Abstract

**Background:**

The Charlson and Elixhauser Comorbidity Indices are the most widely used comorbidity assessment methods in medical research. Both methods are adapted for use with the International Classification of Diseases, which 10th revision (ICD-10) is used by over a hundred countries in the world. Available Charlson and Elixhauser Comorbidity Index calculating methods are limited to a few applications with command-line user interfaces, all requiring specific programming language skills. This study aims to use Microsoft Excel to develop a non-programming and ICD-10 based dataset calculator for Charlson and Elixhauser Comorbidity Index and to validate its results with R- and SAS-based methods.

**Methods:**

The Excel-based dataset calculator was developed using the program’s formulae, ICD-10 coding algorithms, and different weights of the Charlson and Elixhauser Comorbidity Index. Real, population-wide, nine-year spanning, index hip fracture data from the Estonian Health Insurance Fund was used for validating the calculator. The Excel-based calculator’s output values and processing speed were compared to R- and SAS-based methods.

**Results:**

A total of 11,491 hip fracture patients’ comorbidities were used for validating the Excel-based calculator. The Excel-based calculator’s results were consistent, revealing no discrepancies, with R- and SAS-based methods while comparing 192,690 and 353,265 output values of Charlson and Elixhauser Comorbidity Index, respectively. The Excel-based calculator’s processing speed was slower but differing only from a few seconds up to four minutes with datasets including 6250–200,000 patients.

**Conclusions:**

This study proposes a novel, validated, and non-programming-based method for calculating Charlson and Elixhauser Comorbidity Index scores. As the comorbidity calculations can be conducted in Microsoft Excel’s simple graphical point-and-click interface, the new method lowers the threshold for calculating these two widely used indices.

**Trial registration:**

retrospectively registered.

**Supplementary Information:**

The online version contains supplementary material available at 10.1186/s12874-021-01492-7.

## Background

Identification of preexisting clinical conditions or comorbidities is of interest in all types of medical research. The Charlson and Elixhauser Comorbidity Indices are of the most widely used comorbidity assessment methods [1–10] validated on several patient populations like cancer [11], chronic renal failure [12], coronary artery bypass grafting [12], diabetes [12], hip fracture [10, 13–15], and stroke [16]. The Charlson Comorbidity Index (CCI) is a weighted score that accounts for the presence of 19 comorbid diseases [1]. CCI was later adapted for use with administrative data based on the International Classification of Diseases [4]. Later, Quan and his colleagues (2011) provided updated weights for CCI, as treatment of some diseases has improved in time [5]. The Elixhauser comorbidity system was initially developed for administrative data when measuring the presence of 30 comorbidities [17]. Van Walraven and his colleagues (2009) later modified the initial classification system into a single weighted score – Elixhauser Comorbidity Index (ECI) [9]. Later studies have provided different weighting schemes for ECI: Thompson and AHRQ (the Agency for Healthcare Research and Quality, Canada) weights [18, 19].

Currently, available CCI and ECI calculation methods are limited to a few software applications, which can only be operated through a command-line user interface, requiring R, SAS or SQL programming language skills [20–27]. Other available calculators allow measuring only a single patient’s comorbidities at a time [28]. As all CCI and ECI dataset calculators require specific programming-based software, the indices’ accessibility is limited for those who use other software for statistical analyses or have no prior programming experience. Thus, the accessibility of CCI and ECI can be increased by developing new methods using more user-friendly interfaces. Microsoft Excel is a widely used spreadsheet programme, and its graphical point-and-click interface allows use without programming. Thus, this study aims to use Microsoft Excel to develop a non-programming and ICD-10 based dataset calculator for CCI and ECI and to validate its results with R- and SAS-based methods.

## Methods

### Patients

We used real retrospective hip fracture population-wide data from the Estonian Health Insurance Fund. The Estonian Health Insurance Fund organises a national, solidarity-based mandatory health insurance system in Estonia, covering 94% of the population [29]. The hip fracture population was chosen as ICD-10 codes have been found suitable for fracture identification [30], and CCI and ECI have been validated among these patients [10, 13–15, 31]. The inclusion and exclusion criteria were chosen in concordance to multiple other studies [30, 32–35]: (1) age 50 or over; (2) ICD-10 codes S72.0–2 identifying index hip fracture between 1 January 2009–30 September 2017; (3) data validation confirming hip fracture diagnosis and excluding isolated acetabular, pelvic, periprosthetic, isolated greater and lesser trochanter fractures.

### Data validation

**Fig. 1:**
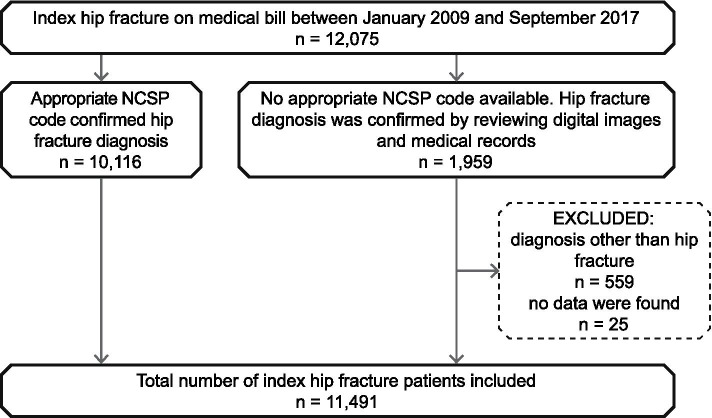
Flowchart showing the validation of hip fracture diagnoses. Abbreviations: NCSP: Nordic-Medico-Statistical Committee’s Classification of Surgical Procedures

The data validation was based on a logic check or the reviewal of patients’ medical information (Fig. 1). Firstly, patients’ Nordic Medico-Statistical Committee’s Classification of Surgical Procedures (NOMESCO) surgical management was reviewed to confirm their hip fracture diagnosis. Following codes confirmed the diagnosis: NFB20, NFB30, NFB40, NFB99, NFB00–9; NFB10–9, NFJ70–3, NFJ60–3, NFJ80–3, NFJ50–3 [36]. If these codes were not available, a patient’s digital images and medical records were reviewed. Two national databases were used to review digital images and medical records: the Foundation of Estonian PACS (an image archiving and communication system database) and the Estonian National Health Information System (https://ap.digilugu.ee/arstiportaal). Uploading medical data to both databases is mandatory by law, particularly since 2010 for medical records and since 2014 for digital images. Digital images were reviewed from January to July 2017 and medical records from January to March 2019. An orthopaedic surgeon and a radiologist reviewed the digital images, and a geriatrician reviewed the medical records. Hip fracture diagnosis was confirmed if one or both of the data sources approved its presence.

### Patients’ comorbidities

Comorbidities were defined as diagnoses coded as ICD-10 at any hospital or outpatient health care claims during a four-year period: at the time of the index HF and during the preceding 4 years. The 4 year preceding period was chosen to avoid under-ascertainment of comorbidities [37]. Finally, a restriction was applied to increase the validity of comorbidity assessment: only ICD-10 codes that appeared at least two times, and at least 7 days apart were included [13, 38].

### Development of excel-based calculator

The Microsoft Excel-based dataset calculator was developed using ICD-10 coding algorithms [4], and different weighting schemes of CCI and ECI, the program’s basic formulae and wide format (Additional file 1). The 10th revision of the International Classification of Diseases was chosen as it is used by more than a hundred countries, including Estonia, and cited in more than 20,000 scientific articles world [4, 39]. The weighting schemes included the original [1] and the updated [5] CCI weights, and van Walraven [9] and AHRQ weights [18]. The calculator also takes into account the hierarchy of comorbidities: milder disease forms are excluded if a more severe one is present. Excel’s basic formulae were used for making the calculator as this makes it simple and flexible for users. It calculates comorbidity scores in two steps. If cell A2 is a patient’s ID and B2 contains her/his diseases as ICD-10 codes, the first step identifies the patient’s comorbidity categories *[=IF (SUM (IF((LEN(B2)-LEN (SUBSTITUTE (UPPER(B2),{“CODE-1”;"CODE-2”; …; “CODE-N”},”“))),1,0)) > 0,1,0)*] and the second step uses the output of the previous step and calculates total score using necessary weights (multiplications) and hierarchical conditions (IF functions) [=(C2*1) + IF(C2 = 0,D2*1,0) + … + IF(M2 = 0,L2*2,0)]. These basic formulae also allow users to edit or adapt the calculator for other weights or versions of the International Classification of Diseases codes. Wide-format, showing one subject per row, was preferred as this is the most used final data structure in statistical analysis. As ICD-10 data is occasionally in long format - one morbidity per row, simple data transformation solutions are included in the calculator’s instructions and in its one-minute instructional video (Additional file 2). Data transformations were done with an Excel’s add-in named Ablebits (www.ablebits.com). The add-in’s functions ‘Merge Duplicates’ (transforms long format to wide format), ‘Merge Cells’ (combines codes from multiple columns into one) and ‘Split Text (splits codes from one cell to multiple columns or rows; transforms wide format to long format) are useful for such purposes. The calculator allows ICD-10 codes to be inserted in any format: lowercase, uppercase, with or without punctuation, and any separators can be used between diagnoses. Finally, the calculator’s ability to identify all ICD-10 codes used in CCI and ECI was tested since the used hip fracture population may not cover all of the diseases used in the indicies. The calculator identified all ICD-10 codes used in the two indices.

### Statistical analysis

Continuous variables were presented as “median (25^th^-75^th^ percentile)” and categorical as proportions. The patients’ Charlson weight comorbidity score and the presence of different diseases were calculated using the Excel-based calculator and the R package “comorbidity” [21] and two SAS macros [40, 41]. The Excel-based calculator was validated by comparing the three methods’ results. The calculators’ processing speeds were compared using the study’s data (multiplicated for larger sample sizes). Excel-based calculator’s processing speed was assessed by running formulae in all columns at once. The analyses were run on a Lenovo T480 laptop released at the beginning of 2018 (i5-8250U 1.6 GHz CPU, 16GB RAM, Windows 10 Enterprise 20H2). Data analyses were done in Microsoft™ Excel™ 365 MSO 16.0.13528.203018 64bit (Microsoft Corporation, Redmond, Washington, USA), R 4.0.4 (R Core Team, 2017) and SAS OnDemand for Academics, release 3.8 (Enterprise edition) (SAS Institute Inc., Cary, NC, USA). Adobe Illustrator and Adobe InDesign (versions CC, Adobe Systems, San Jose, CA, USA) and GraphPad Prism (version 7.0, GraphPad Software, Incorporation, San Diego, CA, USA) were used for creating or finalising figures. Wondershare Filmora (version 10.1.20.16(6.0.0..54.8), Wondershare Technology Corporation, South Shenzhen, China) was used for video editing.

## Results

### Patients and their comorbidities

A total of 11,491 patients were included in the study (Fig. 1). Their median age was 81 years (73–87), 72% (8246) were female, and 51% (5883) had an intracapsular fracture. The Excel-based calculator’s results are presented in two tables: the original and the updated weight CCI scores in Table 1; and AHRQ and van Walraven weight ECI scores in Table 2.

**Table 1:** Original and updated Charlson comorbidity scores and disease categories

Variable	Value
Original Charlson weight score	
Median (25th–75th percentile)	1 (0–3)
Binned estimates	
0	3521 (30.6)
1–2	4841 (42.1)
3–4	2289 (19.9)
≥ 5	840 (7.3)
Updated Charlson weight score	
Median (25th–75th percentile)	2 (0–2)
Binned estimates	
0	4495 (39.1)
1–2	4127 (35.9)
3–4	2258 (19.7)
≥ 5	611 (5.3)
Categories	
Myocardial infarction	796 (6.9)
Congestive heart failure	5025 (43.7)
Peripheral vascular disease	1197 (10.4)
Cerebrovascular disease	2477 (21.6)
Dementia	1106 (9.6)
Chronic pulmonary disease	1243 (10.8)
Rheumatic disease	383 (3.3)
Peptic ulcer disease	542 (4.7)
Mild liver disease	174 (1.5)
Diabetes without complications	1242 (10.8)
Diabetes with complications	678 (5.9)
Hemi- or paraplegia	530 (4.6)
Moderate/severe renal disease	465 (4.0)
Any malignancy	1179 (10.3)
Moderate/severe liver disease	36 (0.3)
Metastatic solid tumor	42 (0.4)
AIDS/HIV	1 (< 0.0)

Values are n (%) unless otherwise specified

**Table 2:** AHRQ and van Walraven Elixhauser comorbidity scores and disease categories

Variable	Value
AHRQ Elixhauser score	
Median (25th–75th percentile)	3.0 (0–8)
Binned estimates	
0	4790 (41.7)
1–4	1257 (10.9)
5–9	3181 (27.7)
≥ 10	2263 (19.7)
van Walraven Elixhauser score	
Median (25th–75th percentile)	5.0 (0–10)
Binned estimates	
0	4295 (37.4)
1–4	1187 (10.3)
5–9	2844 (24.7)
≥ 10	3165 (27.5)
Categories	
Congestive heart failure	5025 (43.7)
Cardiac arrhythmias	2373 (20.7)
Valvular disease	357 (3.1)
Pulmonary circulation disorders	152 (1.3)
Peripheral vascular disorders	1197 (10.4)
Hypertension, uncomplicated	2972 (25.9)
Hypertension, complicated	6230 (54.2)
Paralysis	530 (4.6)
Other neurological disorders	1060 (9.2)
Chronic pulmonary disease	1243 (10.8)
Diabetes, uncomplicated	968 (8.4)
Diabetes, complicated	1063 (9.3)
Hypothyroidism	587 (5.1)
Renal failure	465 (4.0)
Liver disease	183 (1.6)
Peptic ulcer disease excluding bleeding	263 (2.3)
AIDS/HIV	1 (< 0.0)
Lymphoma	69 (0.6)
Metastatic cancer	42 (0.4)
Solid tumour without metastasis	1085 (9.4)
Rheumatoid arthritis collagen vascular diseases	414 (3.6)
Coagulopathy	34 (0.3)
Obesity	184 (1.6)
Weight loss	21 (0.2)
Fluid and electrolyte disorders	46 (0.4)
Blood loss anaemia	124 (1.1)
Deficiency anaemia	771 (6.7)
Alcohol abuse	367 (3.2)
Drug abuse	16 (0.1)
Psychoses	254 (2.2)
Depression	1125 (9.8)

Values are n (%) unless otherwise specified. Abbreviations: *AHRQ* the Agency for Healthcare Research and Quality

### Comparison of the two methods

A total of 192,690 Charlson’s and 353,265 Elixhauser’s output values were compared. The Excel-based calculator’s results were consistent, revealing no discrepancies, with the R- and SAS-based methods.

### Processing speed

**Fig. 2:**
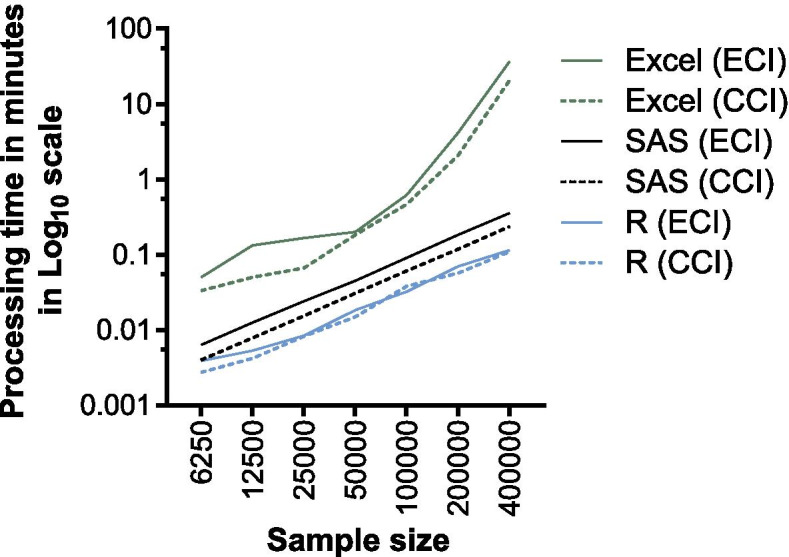
Processing speeds of Excel-based calculator and R- and SAS-based methods with different sample sizes. Abbreviations: CCI: Charlson Comorbidity Index; ECI: Elixhauser Comorbidity Index; SAS – Statistical Analysis Software

The Excel-based calculator performed well with sample sizes of up to 200,000 patients, showing a processing time from 2 s up to 4 min and 10 s (Fig. 2). However, calculating comorbidities for 400,000 patients took 21 min and 32 s for CCI and 36 min and 34 s for ECI with the used hard- and software. In contrast, the R- and SAS-based calculators performed all calculations in less than 22 s.

## Discussion

This study proposes a novel, simple and validated tool for the most used comorbidity indices in medical research – CCI and ECI. The compared methods performed similarly in terms of accuracy, although the new tool has advantages and disadvantages that should be considered. The main advantage of the Excel-based method is its ease of use: comorbidity scores can be calculated by just copying and pasting patients’ identification numbers and ICD-10 codes from one spreadsheet to another. This can be done in a simple graphical point-and-click interface, requiring no coding skills from its user. However, the Excel-based calculator has limitations. Other programming-based methods allow calculating CCI and ECI with earlier versions or adaptions of the International Classification of Diseases: ICD-9, ICD-9-CM, Enhanced ICD-9-CM codes, or ICD-10-CM [5, 20–27]. The new calculator’s processing speed is reasonable with datasets of up to 200,000 patients and relatively capable hardware, taking up to few minutes in total. Still, it may take a considerable amount of time with larger data. This is explained by the calculator’s formulae-based nature, as they are duplicated in millions of spreadsheet cells, requiring a considerable amount of computing power. Computers with better hardware specifications (especially central processing unit’s [CPU] speed, random-access memory [RAM]) and 64bit version Microsoft Excel are therefore recommended for analysing large data. On the other hand, most medical research studies examine smaller sample sizes, large data splitting is always an option, and an hour-long calculation still takes significantly less time than learning to code. Another limitation is that Excel’s spreadsheets are limited to slightly over a million rows. Therefore, large long-format data may require splitting and should be prepared using multiple sheets.

Ultimately, the final choice between using the Excel-based and other methods depends on a user’s skills, preference, available software and needs. All these factors vary among researchers. The new calculator may be useful for users preferring Microsoft Excel to prepare or analyse data, or those who have no programming skills, or whose used statistical software does not have a module for calculating CCI or ECI. Thus, the new simple Excel-based method lowers the threshold for calculating CCI and ECI, making these indices accessible to a broader audience.

## Conclusions

This study proposes a novel, validated, non-programming based method for calculating two of the most used comorbidity indices in medical research - CCI and ECI. The Excel-based calculator allows calculating these comorbidity indices by simply copying and pasting data in a graphical point-and-click interface, thereby lowering the threshold for calculating CCI and ECI. The method may be useful for users preferring Microsoft Excel to prepare or analyse data, or those who have no programming skills, or whose statistical software does not have a corresponding module. The calculator’s slower processing speed is a downside that should be taken into account with very large datasets or less capable hardware or 32bit version of Microsoft Excel.

## Supplementary Information

**Additional file 1.**

**Additional file 2.**

## Data Availability

The study data was not publicly available, but EHIF allowed making anonymized ICD-10 data publicly available. The dataset analysed during the current study is available in the figshare repository, 10.6084/m9.figshare.14046311.

## Abbreviations

AHRQ: The Agency for Healthcare Research and Quality
CCI: Charlson Comorbidity Index
ECI: Elixhauser Comorbidity Index
ICD-9: International Classification of Diseases, 9th revision
ICD-9-CM: International Classification of Diseases, 9th revision, Clinical Modification
ICD-10: International Classification of Diseases, 10th revision
ICD-10-CM: International Classification of Diseases, 10th revision, Clinical Modification
